# Superior Photo-thermionic electron Emission from Illuminated Phosphorene Surface

**DOI:** 10.1038/s41598-019-44823-x

**Published:** 2019-07-16

**Authors:** S. Madas, S. K. Mishra, S. Kahaly, M. Upadhyay Kahaly

**Affiliations:** 1 ELI-ALPS, ELI-HU Non-Profit Ltd., Dugonics ter 13, Szeged, 6720 Hungary; 2 Physical Research Laboratory (PRL), Navrangpura, Ahmedabad, 380009 India

**Keywords:** Applied physics, Electronic properties and materials

## Abstract

This work demonstrates that black phosphorene, a two dimensional allotrope of phosphorus, has the potential to be an efficient photo-thermionic emitter. To investigate and understand the novel aspects we use a combined approach in which *ab initio* quantum simulation tools are utilized along with semiclassical description for the emission process. First by using density functional theory based formalism, we study the band structure of phosphorene. From the locations of electronic bands, and band edges, we estimate the Fermi level and work function. This leads us to define a valid material specific parameter space and establish a formalism for estimating thermionic electron emission current from phosphorene. Finally we demonstrate how the emission current can be enhanced substantially under the effect of photon irradiation. We observe that photoemission flux to strongly dominate over its coexisting counterpart thermionic emission flux. Anisotropy in phosphorene structure plays important role in enhancing the flux. The approach which is valid over a much wider range of parameters is successfully tested against recently performed experiments in a different context. The results open up a new possibility for application of phosphorene based thermionic and photo-thermionic energy converters.

## Introduction

Although, research on layered black phosphorus initiated more than a century ago^1^, exfoliation of phosphorene, an atomically thin two-dimensional (2D) material, from its layered bulk counterpart is experimentally achieved only recently^2,3^. Since these first demonstrations it is being considered a unique addition to the list of emerging 2D materials with multitude of potential applications in nanoelectronics and nanophotonics. While graphene^4,5^, a planar honeycomb all-carbon 2D structure, has pertinence that rely on its exceptional properties such as high carrier mobility and high thermal conductivity^5,6^, the absence of bandgap and its low on-off ratios set limits on its performance^7^. On the other hand, semiconducting 2D transition metal dichalcogenides (TMDs), such as MoS_2_ which have high on-off ratios but lower carrier mobility^8^ compared to graphene^9^, loses out on fast electronic applications.

In contrast, phosphorene with both sufficiently large electronic band gap and high carrier mobility is an ideal candidate system intermediate between graphene and TMDs, for wide ranging opto-electronic applications as well as a new functional component for heterostructure synthesis^10,11^. High carrier mobility up to 1000 cm^2^ V^−1^ s^−1^ ^3^ (comparable with graphene), experimentally demonstrated tunable direct band gap of 2.05–2.20 eV^12^ fuelled massive interest in its application. Depending upon number of layers, phosphorene displays in-direct to direct band gap transition upon going from bulk to monolayer^13^ and its band gap can be further tuned with strain engineering^14^. Due to this tunability in band structure and wide band gap, phosphorene can demonstrate broadband absorption and strong light-matter interaction^15^, making it more suitable than most of the semiconducting TMDs, in the context of optoelectronic applications. Experimental and theoretical works revealing the electronic properties of phosphorene have been extensively performed in the recent past^3,14^. Field effect transistors based on phosphorene has on-off ratios exceeding 10^5^ is demonstrated^3,16^ and its wide ranging applications have been explored in energy conversion and storage devices^17^, spintronics^16,18^, biosensor design^19^ and optoelectronics^3,20,21^. Recently phosphorene is also identified to be a good thermoelectric material. The thermoelectric figure of merit, *ZT* has been predicted to be 0.2–0.7 in doped phosphorene at low temperatures^22^, reaching up to 2.5 at 500 K^23^.

Although diverse aspects of phosphorene have already been probed and studied in detail, its possible application as a photo-thermionic emitter has not been explored until now. But as we would see there are enough reasons motivating investigation along this directions. It is well established that thermoelectric features in solid state devices are connected to thermionic signatures depending on whether electron transport is diffusive or ballistic^24^ and the two formalisms that describe device currents converge when device size is of the order of the mean free path of electrons within. Ultimately both currents can be reduced to the same mathematical form when considered for system size with this length scale^25,26^. Thus strong thermoelectric behaviour might as well indicate interesting possible thermionic features of a solid state material. A natural question thus arises, whether phosphorene can be considered as a new 2D material for efficient thermionic emitter, a feasibility that has already been demonstrated in case of graphene^27–29^. Additionally for graphene it has been shown that emission flux can be enhanced with the help of photon irradiation^30,31^ proving its utility in photo-thermionic conversion schemes. There have been no such precedents for phosphorene, although the previous observation coupled with demonstrated strong absorption features of phosphorene^15^ points in that direction.

In this work, we demonstrate for the first time the potential of black phosphorene as an efficient thermionic emitter and show that its performance can be further enhanced through photon irradiation. We first address the electronic bands, and location of band edges of phosphorene using *ab initio* density functional theory (DFT) based calculations and estimate its Fermi level and work function. Then utilizing our relaxed lattice geometry, atomic structures and energetics we take advantage of the tight-binding (TB) model to define a suitable dispersion relation with optimized tight-binding parameters. Using these results, we establish a formalism to address co-existing and complementing thermionic and photo-thermionic emission from illuminated phosphorene structures. Incorporating the Fermi-Dirac statistics for electrons, taking into account the effect of electron energy redistribution due to thermal agitation via incident radiation and following Fowler’s approach for the electron emission we derive the expressions for the photo-thermionic and thermionic emission flux. The cumulative emission flux is observed to be sensitive to the parametric tuning of the incident radiation and material specifications. Based on the parametric analysis, the photo-thermionic flux is noticed to strongly dominate over its coexisting counterpart thermionic emission flux, at lower surface temperature, and incident wavelength.

## Quantum ***ab initio*** Based Approach to Define Tight Binding Parameter Space for 2D Phosphorene

As a first step, the analysis of atomic and electronic structures of black phosphorene are carried out using DFT, where the properties of a many-electron system can be determined by using spatially dependent electron density obtained from the self-consistent iterative solutions of Kohn-Sham equations^32^.

Here we employ ultrasoft pseudo-potential for the representation of the valence states within generalized gradient approximation (GGA) scheme as proposed by Perdew, Burke, and Ernzerhof (PBE) for the exchange and correlation energy^33^, as implemented in quantum espresso (QE)^34^ package. These choices of pseudopotentials have been tested to be appropriate for dealing with this particular system^35,36^, and many other 2D materials in general^37,38^. While performing Heyd–Scuseria–Ernzerhof (HSE) hybrid functional calculations are known to provide better band gap, HSE band gaps actually scales linearly with PBE band gaps^35^. Hence PBE results may act as efficient descriptors for more expensive HSE calculations, as well as the trends suggested by the experimental results.

**Figure 1:**
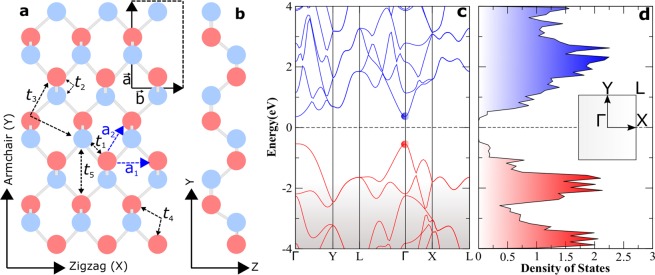
Monolayer black phosphorene structure: (**a**) top view with different tight binding hopping energies *t*_*i*_, (**b**) side view. Red and blue balls represent phosphorous atoms in two different layers. Black rectangular box represents the unit cell structure. (**c**) Electronic band structure corresponding to relaxed crystal, and (**d**) corresponding densities of electronic states (DOS). Inset shows irreducible Brillouin zone with high symmetry points of phosphorene. Fermi level *E*_*f*_ is shifted to 0 in this figure.

We test the convergence of total energy of electrons with respect to *k*-points, energy cutoff and other important parameters and finally chose a plane-wave basis set with an energy cutoff of 60 Ry. The crystal structure is fully relaxed until the final force exerted on each atom reaches below 0.001 eV/Å and an electronic energy convergence threshold of 10^−6^ eV is achieved. For the Brillouin zone integration, we used the *k*-point sets generated by the 12 × 12 × 1 Monkhorst-Pack^39^ *k*-point mesh. Electronic bands are plotted along the high symmetry directions Γ → *Y* → *L* → Γ → *X* → *L*. The puckered honeycomb structure of monolayer black phosphorene is simulated with an in-plane rectangular unit cell (Fig. 1a). To avoid spurious interactions between one unit cell and its successive periodic images in the z-direction, fairly large vacuum of 16 Å is created^40^. Overall our DFT-based results represent quite well the phosphorene relaxed structure^41,42^ and reproduces important features and trends in its band structure accurately.

While DFT calculations provide the full band structure for the relaxed system, simplified analytical relations for the energy dispersion relations are more convenient to use and often give an intuitive understanding of the parametric trends in the final calculation. TB models often achieve this objective and these approaches are considered as stationary approximations to self-consistent DFT calculations^43^. However, TB models have their own limitations and fail to represent complex systems such as structures with impurities, or highly localized electrons (such as in strongly correlated materials). Nonetheless, it is demonstrated that for pristine phosphorene this method can reasonably describe the bands around the Fermi level, if the TB parameters which are functions of interatomic distances, are obtained by directly using DFT band structure^44^. Thus a suitable TB model incorporating the DFT results for phosphorene can be efficiently used for further usage in the estimation of emission currents in our case.

The effective tight-binding Hamiltonian of planar monolayer pristine phosphorene structure^44^ can be expressed as,

1 $$H=\sum _{(i,j)}\,{t}_{ij}{c}_{i}^{\dagger }{c}_{j}=\sum _{i}\,{\varepsilon }_{i}+\sum _{i\ne j}\,{t}_{ij}{c}_{i}^{\dagger }{c}_{j}$$

The summation runs over all the lattice sites *i*(*j*) of the phosphorene unit cell. Here, *ε*_*i*_ is the on-site total energy of the valence electrons at site *i*, *t*_*ij*_ represents the hopping parameter corresponding to the electron transfer energy between *i*^*th*^, and *j*^*th*^ sites. ${c}_{i}^{\dagger }$ (*c*_*j*_) is the creation (annihilation) operator of electrons on the site *i*(*j*). In 2D (x-y plane), electrons in phosphorene are demonstrated to be confined by the parallel energy dispersion *E*_*t*_(*k*_*x*_, *k*_*y*_)^45^. In addition for monolayer phosphorene, it is reasonable to neglect the out-of-plane hopping parameters and restrict all hopping interactions to those in-plane. Since phosphorene has four atoms per unit cell (see Fig. 1a), the energy dispersion should be described by a four band model, within the above mentioned TB framework. Rudenko *et al*. proposed that five hopping integrals^44^ to the nearest and also next-nearest neighbours (i.e., *t*_*ij*_ = *t*_1_, *t*_2_, *t*_3_, *t*_4_, and, *t*_5_ as illucidated in Fig. 1a) suffice to describe the energy band structure of phosphorene in the region 0.3 eV above and below the band gap. Note that for monolayer phosphorene, while *i* = 1, *j* varies from 1 to 5. Hence for simplicity the tight binding parameters are presented with single index (1–5). Taking into account the *C*_2*h*_ point group invariance in the phosphorene crystal, through an unitary transformation, the Hamiltonian in Eq. (1) can reduce to simpler (2 × 2) block matrix in the momentum space, and can be written as

2 $$H={\sum }_{{\bf{k}}}{c}^{\dagger }({\bf{k}})\,{\hat{H}^{\prime} }_{k}\,c({\bf{k}}),$$

where

$${\hat{H}^{\prime} }_{{\bf{k}}}=(\begin{array}{cc}{T}^{4}({\bf{k}}) & {T}^{0}({\bf{k}})\\ {T}^{0\ast }({\bf{k}}) & {T}^{4}({\bf{k}})\end{array}),$$

The matrix elements *T* ^0^(**k**) and *T*^4^(**k**) in the momentum space **k** = (*k*_*x*_, *k*_*y*_) are described by,

3 $$\begin{array}{c}{T}^{0}({\bf{k}})={t}_{1}\mathrm{(1}+{e}^{-i{\bf{k}}\cdot {{\bf{a}}}_{{\bf{1}}}})+{t}_{{\bf{2}}}{e}^{-i{\bf{k}}\cdot {{\bf{a}}}_{{\bf{2}}}}+{t}_{3}({e}^{-i2{\bf{k}}\cdot {{\bf{a}}}_{{\bf{2}}}}+{e}^{i{\bf{k}}\cdot {{\bf{a}}}_{{\bf{1}}}-i2{\bf{k}}\cdot {{\bf{a}}}_{{\bf{2}}}})+{t}_{5}{e}^{-i{\bf{k}}\cdot {{\bf{a}}}_{{\bf{1}}}+i{\bf{k}}\cdot {{\bf{a}}}_{{\bf{2}}}},\\ {T}^{4}({\bf{k}})=2{t}_{{\bf{4}}}[\,\cos \,{\bf{k}}\cdot {{\bf{a}}}_{{\bf{2}}}+\,\cos \,{\bf{k}}\cdot ({{\bf{a}}}_{{\bf{1}}}-{{\bf{a}}}_{{\bf{2}}})].\end{array}$$

Here **a**_**1**_ and **a**_**2**_ are real space lattice vectors as shown in Fig. 1a. The energy eigen spectrum *E*(**k**) of the Hamiltonian in Eq. (2) is obtained, after diagonalization, analytically from the solutions of the following characteristic equation,

4 $$|\begin{array}{cc}E({\bf{k}})-{T}^{4}({\bf{k}}) & {T}^{0}({\bf{k}})\\ {T}^{0\ast }({\bf{k}}) & E({\bf{k}})-{T}^{4}({\bf{k}})\end{array}|=\mathrm{0,}$$

5 $${\rm{i}}.{\rm{e}}.,\,{E}_{\pm }({\bf{k}})={T}^{4}({\bf{k}})\pm |{T}^{0}({\bf{k}})|,$$

Here the (±) in the subscript of *E*_±_(**k**) correspond to (±) in the right hand side of Eq. (5). Thus the solutions represent the energy bands of phosphorene close to the Fermi level, and is further utilized in the calculation for electron emission from phosphorene. Note that, for *t*_4_ = 0, the solution becomes, symmetric; i.e., spectral asymmetry in the energy spectrum of phosphorene is a consequence of non-zero hopping *t*_4_, which is responsible for the electron-hole symmetry breaking of the energy spectrum, and anisotropy of the crystal.

## Electronic Band Structure, Frozen Density of States and Work Function

In-plane projection of black phosphorene lattice shows a hexagonal honeycomb structure, with lattice vectors, *a*_1_, and *a*_2_, as shown in Fig. 1a. Unlike graphene, which is a planar layer of carbon atoms, black phosphorene monolayer has two parallel planes with two atoms in each plane, thus four phosphorous atoms per single unit cell. This results in a puckered honeycomb structure with each phosphorous atom covalently bonded to three adjacent atoms as shown in the Fig. 1a (corresponding side view in Fig. 1b). Consequently the system exhibits an anisotropic crystal structure. Our optimized lattice constants, a = 4.62 Å, and b = 3.29 Å are in good agreement with previous theoretical^46^ and experimental works^42^. Electronic band structure and corresponding densities of states (Fig. 1c,d) of phosphorene reveals a direct band gap of 1 eV at Γ point. Wave function analysis reflects that the upper part of the valence bands consists of the bonding 3*p* orbitals, while the lower part of the conduction bands of the anti-bonding 3*p* orbitals, with primarily *p*_*z*_ contribution and some admixture of 3*s* states at Γ point. The reciprocal lattice along with the high symmetry points are shown in the inset of Fig. 1d. Our results for phosphorene band structure is used for comparing the DFT-based and TB-based electronic bands to tune the tight-binding parameters, and also for obtaining the work function of the materials.

**Figure 2:**
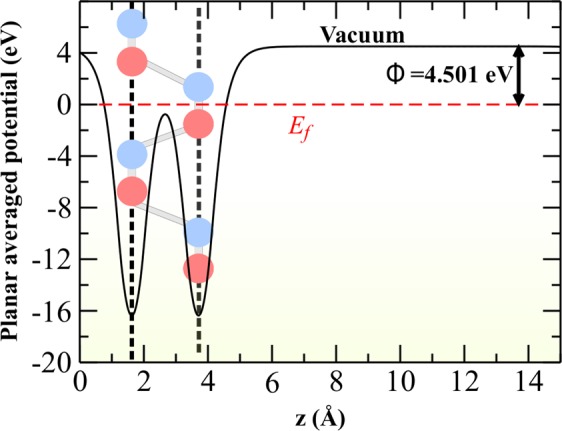
Planar averaged (in x-y plane) potential as a function of height Z from the phosphorene surface (along the normal direction). The phosphorene side view is overlaid on the same plot to emphasize that the location of the dips in the average potential curve (the vertical black dashed lines) coincides with the positions of the atomic planes along Z. The average potential asymptotically approaches the constant vacuum value. Work function is calculated by taking the difference between asymptotic vacuum potential and the Fermi level marked by the horizontal red dashed line for black phosphorene structure from this analysis. *E*_*f*_ is shifted to 0 in this figure.

The work function of monolayer phosphorene, i.e., the energy required to move an electron from the Fermi energy level into vacuum level, is calculated from the difference between the vacuum and Fermi energies, *E*_*vac*_ − *E*_*f*_ (the Fermi energy being defined as the average of the valence-band and conduction-band edges). For this purpose, an infinite array of 2D periodic slabs of phosphorene monolayer is separated by wide vacuum spacing (of 16 Å), so that the electrostatic interactions between two sides of a slab are negligible. The effective spatial distribution of the electrostatic potential is calculated. From the average of this potential in the planes parallel to the surface (as shown in Fig. 2), we obtain the electrostatic potential in vacuum, and hence the work function. The two minima in the potential represents two atomic planes in monolayer phosphorene and about 3 Å above the second atomic plane, the electrostatic potential saturates to a positive asymptotic value. Calculated effective work function is 4.501 eV (see Fig. 2), and is in excellent agreement with previously calculated work function of 4.5 eV^47^.

## Semi-Analytical Formulation of Electron Emission From 2D Phosphorene Structures

In general the analytical model of electron thermionic emission in low dimensional materials has received major attention in recent years. For example specifically for graphene, a Landauer-like model that assumes that electron momentum component lateral to the emission direction is non-conserving^48^ and models that assume momentum conservation^49,50^ have been proposed. All these have enabled identification of universal analytical features in thermionic transport in 2D materials^51^. In our work we introduce a semi-analytical formalism incorporating the results obtained from *ab initio* DFT calculations to investigate the photon assisted thermionic emission behaviour of phosphorene as detailed below.

A closer inspection of the energy spectrum of the two band model (Eq. (5)), reveals that bands with *E*_+_(**k**) has lower energy than bands associated with *E*_−_(**k**). *E*_+_(**k**) bands suffice to describe the major electron and hole bands around the Fermi level and are considered for the electron emission. Thus the parallel dispersion *E*_*t*_(**k**) assumes a further simplified form of the two band energy spectrum (Eq. (5)),

6 $${E}_{t}({\bf{k}})={E}_{+}({\bf{k}})={T}^{4}({\bf{k}})+|{T}^{0}({\bf{k}})|,$$

From the comparison of the DFT band structure with the TB bands, we adopt adjusted values of the TB parameters; *t*_1_ = −1.22 eV, *t*_2_ = 3.665 eV, *t*_3_ = −0.205 eV, *t*_4_ = −0.105 eV, and *t*_5_ = −0.055 eV. Eq. (6) along with these *t*_*i*_ parameters is further used in the analytical formalism for thermionic and photon-assisted thermionic emissions from irradiated phosphorene surface.

**Figure 3:**
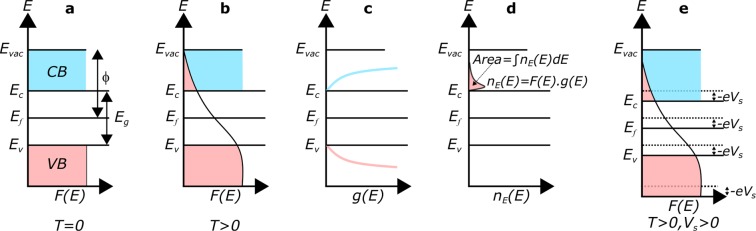
Schematic representation of Fermi-Dirac (FD) distribution and effective number of available electrons under different temperature and biasing conditions. (**a**) Fermi-Dirac distribution and energy diagram at absolute zero, with filled valence bands (VB) and empty conduction bands (CB). *E*_*v*_ represents the VB maximum and *E*_*c*_ represents the CB minimum. *E*_*f*_ is the Fermi level. *E*_*g*_(=*E*_*c*_ − *E*_*v*_) is the band gap energy. *ϕ* is the energy required for a electron from *E*_*f*_ to escape in to vacuum energy level (*E*_*vac*_), above which the electron is considered to be free. (**b**) Modified Fermi-Dirac distribution at a finite temperature T with the distribution tail extending to CB (pink shade). Here a fraction of electrons in VB gains energy and populate CB. (**c**) Density of states (DOS) *g*(*E*) near the band edges. (**d**) Probabilistic occupancy of total number of electrons in CB. (**e**) Modification in energy distribution under a finite positive surface potential (V_*s*_ ≥ 0), when all energy bands are downshifted by −eV_*s*_, resulting in enhanced electron population in CB as compared to (**d**) at the same T.

The radiation flux incident over phosphorene surface serves a dual purpose: (i) a fraction of absorbed energy is utilized in rising of surface temperature through collisional heating, while (ii) rest of the photon flux induces direct photoemission of electrons. The electrons in the phosphorene bands are characterized by Fermi-Dirac (FD) distribution. The schematic in Fig. 3a–e illustrates the different scenarios under this simplistic scheme. Panel (a) in Fig. 3 represents the energy diagram for an unbiased surface, at 0 K. In this case, the electrons are filled upto the maximum of valence band (VB), and *ϕ* is the work function of the material. In going from absolute zero (T = 0 K) to finite temperature (T > 0 K in Fig. 3b), the high energy tail of the electron population is extended beyond *E*_*f*_. The electron population that overcomes the barrier height gets emitted. The available number of electrons or the density of states near the band edges is represented in the panel c. The probability that electrons are occupied in the available states depends on the electron energy distribution and DOS. This scenario is shown in the panel d. There are two ways in which the phosphorene surface can be biased. Firstly, the surface might be biased due to application of an external potential. Secondly, the freely suspended phosphorene structure might acquire a finite positive potential in the steady state scenario due to a dynamic equilibrium between continuous emission of electrons and further recollection over its surface. In our consideration we deal with an infinite (in x-y) phosphorene sheet and this scenario is more pertinent. In either case this effect is lumped into a potential V_*s*_ and the corresponding situation is indicated in panels c and d in Fig. 3. Under a finite positive potential, the whole energy structure moves down by energy - eV_*s*_, as indicated in the last panel of Fig. 3. Note that this applied potential V_*s*_ tunes the effective barrier height for emission, as V_*T*_ = *ϕ* + V_*s*_. All temperature dependent effects appear through the tuning of the FD distribution in the following analysis.

The momentum distribution of electron flux, impinging normally on the top layer surface of phosphorene (at z = 0) from inside and available for emission, having total energy between *E*_*t*_ and (*E*_*t*_ + *dE*_*t*_) and normal energy (along axis ẑ, normal to the surface) between *E*_*z*_ and (*E*_*z*_ + *dE*_*z*_) can be written as^30,52–55^

7 $${d}^{3}n=(\frac{\hslash {k}_{z}}{m})(\frac{1}{2{\pi }^{2}})F(E)d{k}_{x}d{k}_{y}d{k}_{z}=(\frac{{k}_{B}T}{2{\pi }^{2}\hslash {a}_{0}^{2}})F(E)d{k}_{x}d{k}_{y}d{\varepsilon }_{z},$$

where, *F* = *F*_*FD*_ = [1 + exp[*ε*_*z*_ + *ε*_*t*_ − *ε*_*f*_]]^−1^ refers FD distribution of the electrons, *k* = *ka*_0_, *ε*_*t*_ = *E*_*t*_/*k*_*B*_T, ${\varepsilon }_{z}={E}_{z}/{k}_{B}{\rm{T}}={\hslash }^{2}{k}_{z}^{2}\mathrm{/2}\,m{k}_{B}{\rm{T}}$, *ε*_*c*_ = *E*_*c*_/*k*_*B*_T, Γ^0,4^(**k**) = *T* ^0,4^(**k**)/*k*_*B*_T, *ε*_*f*_ = *E*_*f*_/*k*_*B*_T, *E*_*f*_ refers to Fermi energy level, *a*_0_ is the interatomic distance between consecutive layers, ℏ and *k*_*B*_ correspond to reduced Planck’s and Boltzmann’s constants, respectively, and T is the temperature of the electron emitting surface. In obtaining Eq. 7 we have substituted (*ℏk*_*z*_/*m*)*dk*_*z*_ by *ℏ*^−1^*dE*_*z*_ making use of group velocity relation *ℏk*_*z*_/*m* = *dE*_*z*_/*dk*_*z*_.

Consider now that the phosphorene sheet is illuminated by a uniform radiation flux with photon frequency *ν* and normalized energy *ε*_*ν*_ = *hν*/*k*_*B*_T. In terms of the incident light intensity *I*_*in*_ the incident photon flux is given by Λ(*ν*) = *I*_*in*_/*hν*. For an incident radiation power *P*_*L*_ over a finite spot size *σ*_*s*_ one has to use *P*_*L*_ = *σ*_*s*_*I*_*in*_. According to Fowler’s theory^56^, an electron hitting the surface has a probability *β*(*ν*)Λ(*ν*) per unit time of enhancement of normal energy *ε*_*z*_ by absorption of a photon, when Λ(*ν*) photons are incident per unit time per unit surface area. In the quasi-continuum model the total flux of electrons available for normal energy enhancement *n*_*t*_ may be obtained by integrating Eq. (7) over the energy space with normal energy *ε*_*z*_ upto the Fermi level (0, *E*_*f*_). The absorbed fraction *α* of the incident radiation flux (*I*_0_ = *αI*_*in*_) thus goes into *β*(*ν*)Λ(*ν*) fraction of the electron flux that is available for emission. Further, the parameter *β*(*ν*) which effectively leads to the redistribution of the electrons to the higher energy states can be determined by equating the factor *β*(*ν*)Λ(*ν*) of the total electron flux (*n*_*t*_) available for the emission with absorbed incident photon flux as *β*(*ν*)Λ(*ν*)*n*_*t*_ = (*I*_0_/*hν*) ⇒ *β*(*ν*)*n*_*t*_ = *α*. Part of this absorbed energy is (a fraction *μ*) consumed in lattice thermalization to increase its surface temperature while rest of it (fraction 1 − *μ*) is utilized in the process of electron emission. The above description implies that *f*_*P*_ ≡ (1 − *μ*)*β*(*ν*)Λ(*ν*) fraction of the electron flux contributes in photoemission while rest of the fraction *f*_*T*_ ≡ 1 − (1 − *μ*)*β*(*ν*)Λ(*ν*) leads to thermionic emission of electrons.

Next we analyze the two aforementioned cases separately. For usual thermionic emission (*ε*_*z*_ > *ε*_*c*_ with *ε*_*t*_ substituted from Eq. (6)), the momentum distribution associated with the electrons inside sheet can be expressed as,

8 $${d}^{3}{n}_{Th}\,=\,{f}_{T}(\frac{{k}_{B}{\rm{T}}}{2{\pi }^{2}\hslash {a}_{0}^{2}}){\mathrm{[1}+\exp ({\varepsilon }_{z}-{\varepsilon }_{f})\exp [{{\rm{\Gamma }}}^{4}({\bf{k}})+Abs[{{\rm{\Gamma }}}^{0}({\bf{k}})]]]}^{-1}d{k}_{x}d{k}_{y}d{\varepsilon }_{z},$$

where, *n*_*Th*_ is the thermionic emission flux.

The momentum distribution of the electrons after absorbing a photon resulting in photo-enhanced normal energy ${\varepsilon ^{\prime} }_{z}={\varepsilon }_{z}+{\varepsilon }_{\nu }$ and transverse momenta ${k^{\prime} }_{x}={k}_{x},{k^{\prime} }_{y}={k}_{y}$, can be obtained by expressing {*ε*_*z*_, *k*_*x*_, *k*_*y*_} in Eq. (8) in terms of $\{{\varepsilon ^{\prime} }_{z},{k^{\prime} }_{x},{k^{\prime} }_{x}\}$ and can be written as^57^

9 $${d}^{3}{n}_{Ph}\,=\,{f}_{P}(\frac{{k}_{B}{\rm{T}}}{2{\pi }^{2}\hslash {a}_{0}^{2}}){\mathrm{[1}+\exp ({\varepsilon }_{z}-{\varepsilon }_{f}-{\varepsilon }_{\nu })\exp [{{\rm{\Gamma }}}^{4}({\bf{k}})+Abs[{{\rm{\Gamma }}}^{0}({\bf{k}})]]]}^{-1}d{k}_{x}d{k}_{y}d{\varepsilon }_{z},$$

where, *n*_*Ph*_ is the photo-thermionic emission flux. For simplicity we have omitted the primes in Eq. (9). The flux coming out due to thermionic/photo-thermionic emission may be obtained by integrating above expressions (Eqs (8) and (9)) over adequate boundaries in **k**-space and effective surface potential barrier (*ε*_*z*_). Considering the periodic nature of the lattice vectors in parallel dispersion, the integration limits over **k** may be chosen such that it occupies the maximum dimension along X and Y directions in **k** space; for example **k** = 2*π*/(**a**_1_, **a**_2_)_*min*_.

Note that, in order to avoid the effect of tunneling and to capture only the photo-thermionic effects from a suspended phosphorene layer (under dynamic equilibrium), we consider positive surface potential (V_*s*_ ≥ 0). In this case, thermionic/photo-thermionic emission occurs only for *ε*_*z*_ > *ε*_*c*_ − *υ*_*s*_, where *υ*_*s*_ = −eV_*s*_/*k*_*B*_T. The net thermionic/photo-thermionic flux thus may be expressed as^30^

10 $${n}_{Th,Ph}=\int \,{d}^{3}{n}_{Th,Ph}={\int }_{{\varepsilon }_{c}-{\upsilon }_{s}}^{\infty }\,({\int }_{-{k}_{x0}}^{{k}_{x0}}\,{\int }_{-{k}_{y0}}^{{k}_{y0}}\,{d}^{3}{n}_{Th,Ph}d{k}_{x}d{k}_{y})d{\varepsilon }_{z}.$$

Thus, the corresponding thermionic/photo-thermionic emission current density *J*_*Th*,*Ph*_ can be evaluated from the flux given by Eq. (10) as, *J*_*Th*,*Ph*_ = *en*_*Th*,*Ph*_. The thermionic/photo-thermionic emission flux from unbiased surface can be obtained by setting *υ*_*s*_ = 0 in the above expression (Eq. (10)).

## Results and Discussion

### Thermionic current density and its dependence on anisotropy

**Figure 4:**
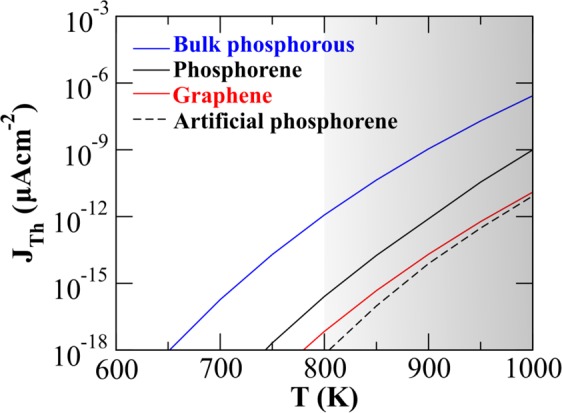
Thermionic emission current as a function of surface temperature. The blue curve refers to usual bulk (parabolic dispersion), red curve refers to 2D graphene (linear parallel dispersion), while black curve refers to 2D phosphorene (2D anisotropy parallel dispersion) while the broken black curve refers to 2D isotropic dispersion of phosphorene. Here the shaded region indicates where sublimation process in phosphorene would play a role.

In Fig. 4, we compare the thermionic emission flux from black phosphorene to that from its bulk counterpart, and also from graphene, operating at a finite temperature (600–1000 K). The three cases are primarily differentiated by their dispersion relations. The phosphorene bulk counterpart is considered as stacking of phosphorene multilayers and its work function (*ϕ* = 4.03 eV) has been taken from recent experimental work^58^. If we neglect the anisotropy of phosphorene structure (assuming *t*_4_ = 0, and thus a symmetric energy spectrum), the emission current (black dashed line) is reduced by almost two orders of magnitude. Thus anisotropy is anticipated to play an important role. In fact when compared with the flux from monolayer graphene (considering linear parallel dispersion), an established material utilised for efficient thermionic conversion, anisotropic phosphorene results in higher thermionic current, particularly at higher temperature regime; this anisotropy feature may be exploited in fabricating efficient phosphorene based cathodes in thermionic conversion schemes. Physically in all cases, the rise in the surface temperature increases the fraction of high energy electrons in the population density distribution function and subsequently the emission current (Fig. 4).

We note that the stability of thin layers of black phophorus (BP) depends on ambient oxidation conditions^59–63^ as well as on the operating temperature. For experiments that can be undertaken in vacuum oxidation can be avoided altogether. Thermal degradation of phosphorene has been experimentally observed to begin around ~700 K^64,65^ in vacuum (in contrast to ~883 K^66–68^ for bulk BP sublimation temperature). On the other hand in presence of ambient nitrogen, sublimation of few layer thin BP is reported at a comparatively lower temperature^69,70^. Nonetheless, phosphorene degradation under ambient conditions can be overcome by adopting different mitigation strategies^71,72^ without significantly affecting phosphorene properties^73^ whereas the operating temperature and thermal stability can be further boosted^74^ through several means, such as through formation of heterostructures with graphene^75^. With this in mind, we calculate the thermionic emission flux upto temperatures elevated beyond the current thermal tolerance of BP (the shaded region in Fig. 4). While the model predictions are experimentally pertinent within the un-shaded region of Fig. 4, the shaded region lets us check the consistency of our theoretical prediction. This confirms that both the results for graphene and artificial phosphorene approach each other with increasing temperature, where the thermionic contribution for phosphorene retains its overall higher value.

### Dependence of emission current on work function

**Figure 5:**
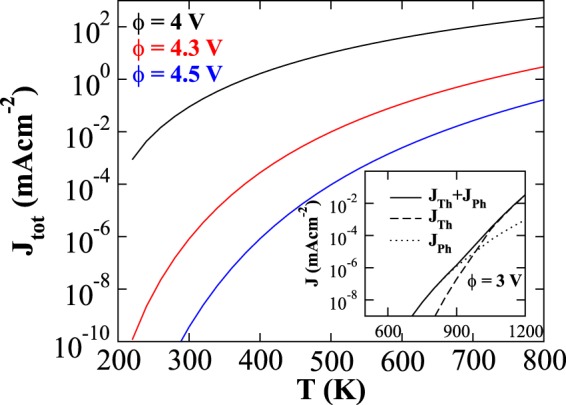
Total emission flux *J*_*tot*_(=*J*_*Th*_ + *J*_*Ph*_) as a function of surface temperature for different values of work function, 4 V (black curve), 4.3 V (red curve), and 4.5 V (blue curve). Wavelength of the irradiated photon is considered to be 300 nm. In inset, for comparison, total current (solid curve), thermionic (dashed curve), and photoelectric emission current (dotted curve) are shown. It depicts flux as a function of surface temperature which is calculated by considering work function as 3 V and wavelength of the irradiated photon as 800 nm.

Total emission flux *J*_*tot*_(=*J*_*Th*_ + *J*_*Ph*_) for phosphorene as a function of varying surface temperature is shown in the Fig. 5, for *λ* = 300 nm. For comparison, three different work function values have been considered. The flux increases with decreasing work function, since less energy would suffice for electron emission. Photo-thermionic emission flux strongly dominates over the thermionic flux; *J*_*Th*_ becomes comparable to *J*_*Ph*_ only at higher temperature regime (see the inset). However, we note that the temperature (inset) at which this predicted transition takes place, sublimation process ensues and the thermal stability of pristine phosphorene degrades, as discussed before, implying that photo-thermionic processes dominate for all practical purposes.

As formulated, the photo-thermionic effect is characterized by thermally agitated modification in momentum distribution function of electrons and comprises of coexisting photon aided thermionic and direct photoemission phenomenon. With decreasing wavelength of incident radiation, direct photoemission naturally grows over the other part. The significant flux at lower temperature (200–500 K) actually promotes photon irradiated phosphorene for photo-thermionic converters for utilizing moderate temperature industrial wastes. The magnitude of the fluxes of course depends on the work function (tunable through materials engineering) and is more pronounced for lower values.

### Variation of photo-thermionic emission flux with surface bias

**Figure 6:**
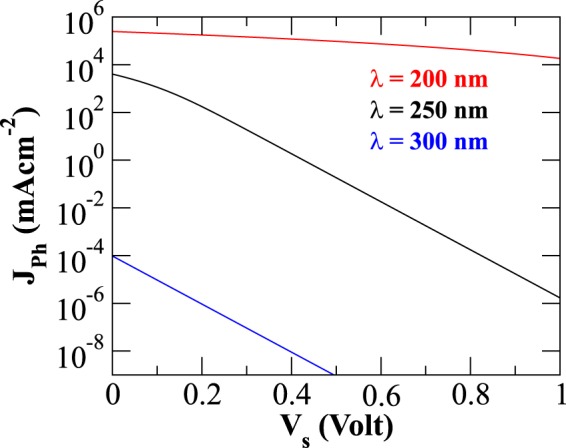
Photo-thermionic emission current of irradiated phosphorene (at T = 500 K) as a function of varying positive potential is shown. Incident light of three different wavelengths, 200 nm (red curve), 250 nm (black curve), and 300 nm (blue curve) are considered here for comparison.

Next, we examine the effect of finite positive potential on the photo-thermionic emission, as illustrated in the Fig. 6. The photoemission flux depends significantly on photon energy and decreases with increasing wavelength; this nature may be attributed to small shift in momentum distribution of agitated electron population available for emission. Although under ambient air conditions it has been demonstrated that changing the wavelength of light from 280 nm to 1050 nm reduces the stability of thin BP layer due to reactive oxygen species^76^, such effects do not arise for operation in vacuum. The positive potential over surface leads to enhanced potential barrier for the emitting electrons as they experience Coulomb attraction, resulting in reduced flux.

**Figure 7:**
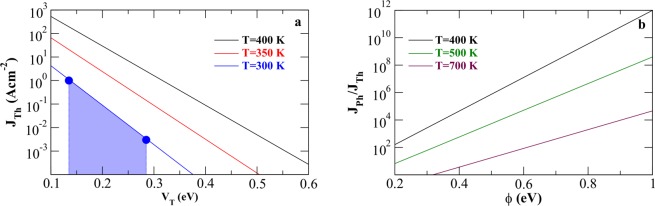
Comparison of the analytical estimates of emission current with experimental data (**a**): current as a function of tuned barrier height. The blue dots represents the experimental data. The ratio of photo-thermionic (*J*_*Ph*_) to thermionic current (*J*_*Th*_), as a function of (**b**) effective barrier height (or, tuned work function) *ϕ*. Wavelength of the irradiated photon is considered to be 300 nm.

Photo-thermionic flux (*J*_*Ph*_) is found to be much stronger than the thermionic flux (*J*_*Th*_) (by some orders of magnitude), for varying range of tuned barrier height (effective work function), as shown in Fig. 7b. Dominance of *J*_*Ph*_ over *J*_*Th*_ increases monotonically with increasing *ϕ* (see Fig. 7b, for *λ* = 300 nm). However, with rise in the operating temperature T, higher population of electron density would be available for thermionic emission; this leads to the decay of the ratio *J*_*Ph*_ and *J*_*Th*_ with increasing T (see Fig. 7b). For large *ϕ* values, low availability of high energy electron population near the vacuum level (V = 0, as in Fig. 3), significantly reduces both the constituent currents, in a way that their ratio change becomes merginal. The ratio of *J*_*Ph*_ and *J*_*Th*_ is also sensitive to incident *λ*, for different operating temperatures and effective barrier height *ϕ*. With increase in incident energy (reducing *λ*), more electrons emit through photo-thermionic mechanism, thereby enhancing *J*_*Ph*_ to *J*_*Th*_ ratio. For phosphorene operating at T = 500 K, direct photoemission becomes significant for *λ* ≈ 500 nm radiation with respect to thermionic flux (ratio of *J*_*Ph*_ and *J*_*Th*_ ≈ 10^10^) while for higher *λ* photon aided thermionic flux is prominent.

### Comparison with experiments

Recent experimental investigations on few layer phosphorene^21^ provides an opportunity to test the prediction of our analytical model against measured values. In these works the authors have primarily focused on the electronic transport characteristics of phosphorene based field effect transistors (FETs). However the results also unravel the thermionic emission aspects relevant to the present study. For instance, Fig. 1b in^21^ representing the transfer characteristics of monolayer phosphorene flake (≈2 × 10 *μm*) operating at room temperature (300 K) under flat band condition (|*V*_*F*_| > |*V*_*BG*_|) reveals a domain where the measured device current (*I*_*DS*_) is purely thermionic, showing a log-linear dependence on the gate bias voltage (*V*_*BG*_). This dependence is shown to be valid within the current voltage range (*V*_*F*_, *I*_*F*_) to (*V*_*M*_, *I*_*M*_), where the subscripts *F* and *M* refer to the flat band condition and the minimum current points respectively. From Fig. 1b in^21^ one gets (*I*_*M*_, *I*_*F*_) ≈ (0.0003 − 0.1) *μA*/*μm* and (*V*_*M*_, *V*_*F*_) ≈ (0, 1.8) V corresponding to a slope, *γ* = Δ*ln*(*I*_*DS*_)/Δ(*V*_*BG*_/*β*) ≈ 25, where *β* is the band movement factor (≈15 in this operating regime^21^) which determines the band movement due to applied gate voltage as ${\rm{\Delta }}\varphi \,=\,({V}_{F}-{V}_{M})/\beta \approx 0.12$. Although these results are relevant to phosphorene in FET mode of operation, the fact that both *I*_*F*_ and the slope *γ* are very weakly dependent on drain bias *V*_*DS*_ enables a direct comparison with thermionic flux calculated using Eq. (10)

Taking the lateral cross section (*σ* ≈ 2 × 10 *μm*) and channel width *l* ≈ 2 *μm* into account, current estimate may be read as (*l* × *I*/*σ*) ≈ (0.003 − 1.0) Acm^−2^. Figure 7a displays the calculated thermionic emission current from an uncharged phosphorene monolayer surface as a function of tuned barrier height (*V*_*T*_) for different values of operating temperatures. The shaded region in Fig. 7a (for T = 300 K) corresponds to the experimental conditions, mentioned above. The calculated slope *γ* (≈21) is in reasonable agreement with measurement. Further, we calculate the rigid band movement corresponding to the (*I*_*M*_, *I*_*F*_) i.e. *V*_*T*_(*I*_*F*_) − *V*_*T*_(*I*_*M*_) = 0.14 V which reproduces well the measured Δ*ϕ*. This suggests the validity of our approach in the estimation of electron emission flux from phosphorene.

At this point we would like to mention that depending on the operational conditions, in 2D materials with external contacts, additional effects might play important role. For example, in mechanically exfoliated molybdenum disulfide (MoS_2_) crystals vertical charge conduction mechanism using Fowler-Nordheim formulation have been successfully applied^77–79^. On the other hand, in chemical vapour deposition grown pyramidal-structured MoS_2_ flakes it has been shown that Richardson-Schottky effect^80^, i.e. the lowering of interface potential maximum due to the presence of image charge effect, plays a role^81^. The role of such effects in phosphorene which has shown its promise as photo-thermionic emitter in this study needs to be seriously investigated in future. Here the emission currents in Eqs (8) and (9), do not incorporate the exact Schottky barrier profile^82^ or the image charge effects explicitly. However we implicitly consider such effects lumped within the potential *V*_*S*_ in evaluation of currents which gives a tuned barrier height for emission, *V*_*T*_ = *ϕ* + *V*_*S*_ (as in Fig. 7, for comparison with experiment).

## Conclusions and Outlook

In this work we demonstrate that novel 2D material phosphorene has the potential to be a good photo-thermionic electron emitter. To achieve this we develop a procedure to calculate the appropriate emission current. The theoretical approach is based on semi-analytical modelling to calculate thermionic emission flux and prescribes how the flux can be modified and enhanced by photon irradiation and model, which is valid for dynamical equilibrium conditions, is verified against experimental observables. Our results suggest that anisotropic energy dispersion of phosphorene results in higher emission flux and a superior emission current when compared to those from graphene. This makes it an efficient candidate for photo-thermionic emission, and thermionic emission-based energy conversion technology. Our calculations are based on adiabatic or continuous irradiation conditions which is sufficient to emphasize the main features of the process and also relevant to many experimental scenarios as exemplified before. For example, the current approach provides a fundamental understanding of photo-thermionic behaviour of 2D phosphorene with signature features that matches remarkably well with experimental results.

The next step would be to adapt the approach for laboratory scale coherent intense optical drivers which are rich in spatio-temporal features^83,84^ and provide the opportunity of complex interaction scenarios. Theoretically dealing with these non-adiabatic spatio-temporally dependent aspects is challenging and would need incorporation of higher order complexity in our approach and constitutes our next aim. In the light of new upcoming state of the art ultrashort laser facilities like ELI-ALPS^85–87^, novel materials like phosphorene might open up new perspectives in potential high yield ultrafast emissions. This work provides the very first theoretical basis to explore this direction.
